# Case report: Reversible punctate inflammatory foci in the corpus callosum: A novel radiological finding of CAR T-cell therapy-related neurotoxicity

**DOI:** 10.3389/fneur.2023.1125121

**Published:** 2023-02-07

**Authors:** Umberto Pensato, Chiara de Philippis, Flavio Pistolese, Daniele Mannina, Simona Marcheselli, Letterio S. Politi, Armando Santoro, Stefania Bramanti

**Affiliations:** ^1^IRCCS Humanitas Research Hospital, Milan, Italy; ^2^Department of Biomedical Sciences, Humanitas University, Milan, Italy; ^3^Humanitas Cancer Center, IRCCS Humanitas Research Hospital, Milan, Italy

**Keywords:** cancer immunotherapy, neuroimaging, brain MRI, cytokine storm-associated encephalopathy (CySE), neuroinflammation, B-cell lymphoma, neuroradiology, hematological cancer

## Abstract

**Introduction:**

Chimeric antigen receptor T-cell therapy-related neurotoxicity is a novel cytokine-mediated neurological syndrome that may present with a broad spectrum of manifestations. Descriptions of novel distinctive features are pivotal to untangling this condition's clinical and instrumental signature in order to inform diagnosis and pathophysiology.

**Case:**

A 27-year-old female patient received anti-CD19 CAR T cells for a refractory primary mediastinal B-cell lymphoma. At 6 days after the infusion, she developed mild ideo-motor slowing, dysgraphia, and drowsiness. Despite specific treatment with dexamethasone, her neurological status progressively worsened to a comatose state within 24 h. EEG and CSF analyses were non-specific, showing background slowing and inflammatory findings. Brain MRI revealed multiple focal punctate areas of T2-weighted hyperintensity localized in the body and isthmus of the corpus callosum. Following the administration of high-dose intravenous methylprednisolone, her neurological status resolved within 48 h. Notably, the follow-up brain MRI did not reveal any abnormalities in the corpus callosum, except for a reduction of fractional anisotropy.

**Conclusion:**

Reversible punctate inflammatory foci of the body and isthmus of the corpus callosum may represent a novel radiological finding of CAR T-cell therapy-related neurotoxicity.

## Introduction

Chimeric antigen receptor (CAR) T-cell therapies are changing the treatment paradigms for B-cell malignancies by achieving durable complete remission in patients refractory to multiple lines of treatment (1). In contrast, the potent anti-lymphoma effect of anti-CD19 CAR T-cell therapy might be hampered by significant toxicities including immune effector cell-associated neurotoxicity syndrome (ICANS) and cytokine release syndrome (CRS) (2–4). ICANS is a cytokine-mediated neurological syndrome that may present with a broad spectrum of manifestations, ranging from mild tremor, dysgraphia, and aphasia to seizures, akinetic mutism, and life-threatening cerebral edema (5–8). The incidence of ICANS is variable according to multiple factors, and in particular, it is higher in the case of infusion of CD28 co-stimulated

products. Descriptions of novel distinctive features are pivotal to untangling this condition's clinical and instrumental signature in order to inform diagnosis and pathophysiology. Most patients present with unremarkable brain MRI, yet a few recurrent neuroradiological features have been recognized (4, 9, 10). Herein, we present a case of CAR T-cell therapy-related neurotoxicity associated with reversible corpus callosum abnormalities, which expand the spectrum of inflammatory neuroradiological abnormalities associated with ICANS.

## Case report

**Figure 1:**
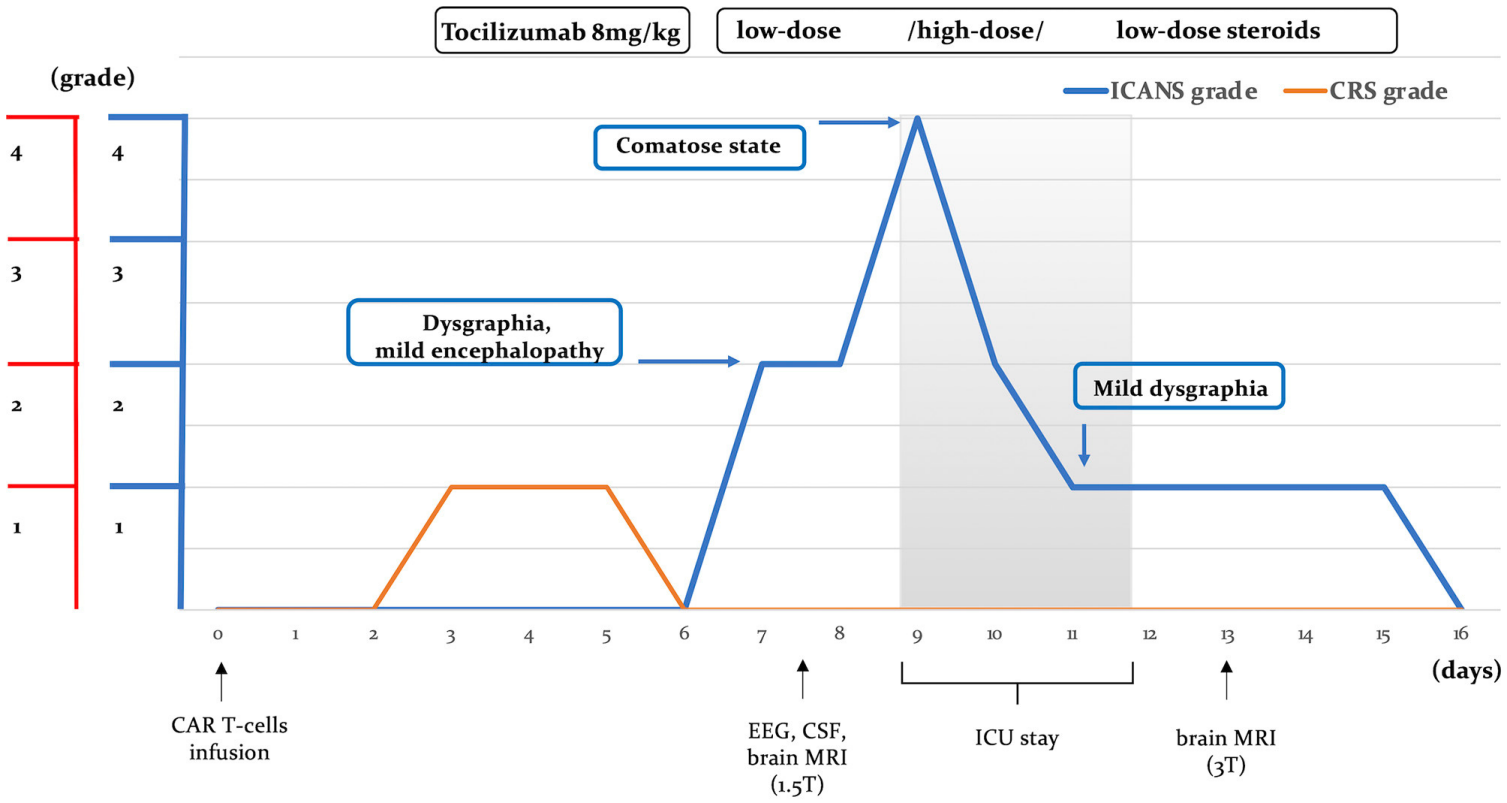
Disease course. Clinical manifestations of neurotoxicity, CRS, and ICANS severity, along with the timing of diagnostic investigations and treatment following CAR T-cell therapy, are illustrated. CRS, cytokine release syndrome; ICANS, immune effector-associated neurotoxicity syndrome; CSF, cerebrospinal fluid; ICU, intensive care unit.

**Figure 2:**
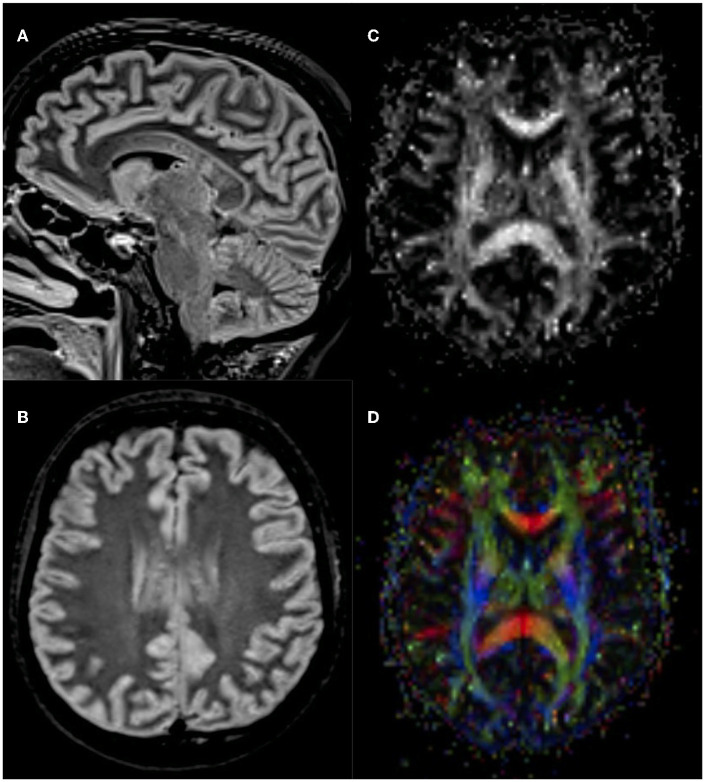
Brain MRI findings. Axial **(A)** and sagittal **(B)** 1.5 Tesla T2-weighted fluid-attenuated inversion recovery (FLAIR) showed multiple focal punctate areas of hyperintensities localized in the splenium and isthmus of the corpus callosum, with no contrast enhancement or diffusion restriction. Follow-up 3 Tesla brain MRI revealed no T2-weighted abnormalities (images not shown). Yet, diffusion tensor imaging (DTI) showed fractional anisotropy reduction within the corpus callosum's body and splenium **(C, D)**.

An otherwise healthy 27-year-old female patient was referred to our center for anti-CD19 CAR T-cell therapy (axicabtagene ciloleucel: axi-cell) for primary mediastinal B-cell lymphoma (PMBCL), stage IIXB at diagnosis for mediastinal bulky, refractory to two lines of standard chemotherapy (Figure 1). Before CAR T-cell infusion, she underwent a 30-Gray mediastinal irradiation as a bridge therapy. After 48 h from CAR T-cell infusion, the patient developed a fever with no associated hypotension or hypoxia (CRS grade 1). In accordance with our local guidelines, for persistent grade 1 CRS, she was treated with tocilizumab (8 mg/kg), resulting in complete resolution of systemic symptoms on day +6. Nevertheless, the following day, she developed mild ideo-motor slowing, dysgraphia, and drowsiness. The clinical picture was consistent with a grade 2 ICANS. A head CT scan was unrevealing, and treatment with dexamethasone (10 mg q6h) was started. EEG revealed frontal intermittent rhythmic delta activity (FIRDA), while cerebrospinal fluid (CSF) analysis showed normal cellular counts and negative comprehensive microbiological investigations, but very high protein levels (320 mg/dL). Despite prompt administration of steroid treatment, at day +9, her neurological status progressively worsened to a comatose state within 24 h (ICANS grade 4); therefore, she was transferred to the intensive care unit (ICU). Brain MRI revealed multiple focal punctate areas of T2-weighted fluid-attenuated inversion recovery (FLAIR) hyperintensity localized in the body and isthmus of the corpus callosum, with no contrast enhancement (Figures 2A, B). No restriction on diffusion-weighted imaging (DWI) or hypointensity on susceptibility-weighted imaging (SWI) was observed within the corpus callosum. Following high-dose intravenous methylprednisolone (1 g daily) administration, her neurological status resolved within 48 h. Neurological examination revealed retrograde amnesia regarding the days spent in ICU and mild dysgraphia that persisted until day +12. Remarkably, the follow-up brain MRI (day +13) did not reveal any abnormalities in the corpus callosum, except for a reduction of fractional anisotropy in the diffusion tensor imaging (DTI) sequences (Figures 2C, D). The patient was re-transferred to the hematology ward, and the corticosteroid therapy was gradually tapered. On day +16, steroid treatment was stopped entirely, and the patient was discharged home. She did not develop any late-onset neurotoxicity, and her lymphoma was still in complete remission at the last follow-up, more than 2 years after CAR T-cell therapy.

## Discussion

We described the case of a young woman affected by refractory PMBCL who displayed undescribed transitory inflammatory abnormalities of the corpus callosum during neurotoxicity related to CAR T-cell therapy. Our patient developed neurological manifestations on the seventh day from axi-cel infusion, subsequently to CRS resolution, and progressed to severe neurotoxicity within 48 h. Accordingly, ICANS median onset time is 4–6 days after CAR T-cell infusion, depending on CRS timing, and a rapid progression within a few h/days has been reported as the typical evolution (7). She presented with dysgraphia, one of the most common early neurological presentations of ICANS, and showed complete resolution following steroid therapy, as often observed in previous cases (6, 7, 11, 12). In terms of instrumental findings, the mild inflammatory CSF findings and EEG frontal abnormalities detected in our patient have been recurrently described (7, 12–14), but her neuroradiological picture has never been described. Brain MRI is usually unremarkable in ICANS, but some neuroradiological abnormalities have been reported, including white matter changes, diffuse cerebral edema, leptomeningeal enhancement, lesions localized in the bilateral thalami, claustrum, and the splenium of the corpus callosum (7, 10, 12). Notably, several of these radiological abnormalities have been consistently observed in cytokine storm-associated encephalopathies beyond ICANS (4). Indeed, a single transitory lesion of the splenium of the corpus callosum is a radiological hallmark of febrile infection-associated encephalopathy disorders, whose underpinning pathophysiology is cytokine-mediated neuroinflammation (4, 15, 16). Nonetheless, the body of the corpus callosum is usually spared in these conditions. Conversely, acquiring acute inflammatory patchy lesions involving the body of the corpus callosum may be observed in other neuroinflammatory disorders, such as primary central nervous system vasculitis, Susac syndrome, and multiple sclerosis (17, 18). Interestingly, a case of Susac syndrome following HIV-associated immune reconstitution syndrome (IRIS) has been observed (19). IRIS is characterized by a paradoxical worsening of clinical conditions resulting from a re-activated immune system, usually following antiretroviral treatment in patients with HIV (19). Therefore, similarities in the underlying pathophysiological mechanisms and clinical findings between the neurological form of IRIS and ICANS may exist.

Notably, at the follow-up brain MRI, no T2-weighted hyperintensity was observable, yet DTI sequences detected microstructural abnormalities related to altered integrity of the white matte fibers of the corpus callosum (20). Collectively, the corpus callosum lesions' localization, morphology, and temporal evolution, along with the persistent microstructural alterations, support an underlying inflammatory nature. The blood–brain barrier disruption and inflammatory demyelination, related to the overarching cytokine-mediate neuroinflammation, are arguably responsible for a spectrum of corpus callosum abnormalities shared by ICANS and other neuroinflammatory conditions.

## Conclusion

Reversible punctate inflammation of the body and isthmus of the corpus callosum may represent a novel radiological finding of CAR T-cell therapy-related neurotoxicity. Descriptions of novel distinctive investigative features are pivotal to defining a neuroradiological signature that would inform diagnosis and pathophysiology.

## Data availability statement

The original contributions presented in the study are included in the article/supplementary material, further inquiries can be directed to the corresponding author.

## Ethics statement

Written informed consent was obtained from the individual(s) for the publication of any potentially identifiable images or data included in this article.

## Author contributions

UP and FP: drafting of the manuscript for content. FP, CP, DM, SM, LP, AS, and SB: revision of the manuscript for content, major role in the acquisition of data, and analysis or interpretation of data. UP, CP, and SB: study concept or design. All authors contributed to the article and approved the submitted version.
